# lncRNA MIR503HG Targets miR-191-5p/PLCD1 Axis and Negatively Modulates Apoptosis, Extracellular Matrix Disruption, and Inflammation in Abdominal Aortic Aneurysm

**DOI:** 10.1155/2023/4003618

**Published:** 2023-05-16

**Authors:** Ye Tian, Xinxi Li, Chao Bai, Zhenwei Yang, Lei Zhang, Jun Luo, Wenbin Zhang

**Affiliations:** ^1^Department of Vascular and Thyroid Surgery, The First Affiliated Hospital of Xinjiang Medical University, Urumqi, 830000 Xinjiang Uygur Autonomous Region, China; ^2^Department of Gastrointestinal Surgery, The First Affiliated Hospital of Xinjiang Medical University, Urumqi, 830000 Xinjiang Uygur Autonomous Region, China

## Abstract

As the most prevalent subtype of aortic aneurysm, abdominal aortic aneurysm (AAA) features the apoptosis, extracellular matrix (ECM) disruption, and inflammation response of vascular smooth muscle cells (VSMCs). Noncoding RNAs (ncRNAs) are crucial factors in AAA progression, while the investigations have not been fully explained. miR-191-5p upregulation is found in aortic aneurysm. However, its role in AAA has not been addressed. This research purposed to excavate the possible and associated molecular axis of miR-191-5p in AAA. In our study, miR-191-5p level was detected to be high in the tissues from AAA patients in comparison with the control group. After miR-191-5p expression was enhanced, cell viability was repressed, cell apoptosis was boosted, and ECM disruption and the inflammation response were fortified. Furthermore, the relationship among MIR503HG, miR-191-5p, and phospholipase C delta 1 (PLCD1) in VSMCs was disclosed via mechanism assays. Decreased MIR503HG lacked the inhibition on miR-191-5p targeting PLCD1, resulting in downregulation of PLCD1, which facilitated the progression of AAA. Thus, targeting MIR503HG/miR-191-5p/PLCD1 pathway will provide an additional method for the cure of AAA patients.

## 1. Introduction

Focal dilatations of abdominal aorta with about 50% bigger than proximal normal segment or thickness more than 30 mm in diameter are defined as abdominal aortic aneurysm (AAA) [1, 2]. According to our knowledge, AAA is life-threatening due to its high morbidity and mortality among adults, especially among the older [3–5]. Unfortunately, the regulatory mechanisms underlying AAA pathogenesis are not well-illustrated, and therefore, effective therapeutic targets are lacking for AAA treatment. Increasing evidence has supported that vascular smooth muscle cells (VSMCs) are able to secrete elastin, an essential element of collagen synthesis and AAA wall remodeling [6, 7]. Therefore, the exploration of the cellular activities in VSMCs may be beneficial to the treatment of AAA.

Noncoding RNAs (ncRNAs) are transcripts that lack open reading frames (ORFs) and cannot encode proteins [8, 9]. Long ncRNAs (lncRNAs) with over 200 nucleotides (nt) and microRNAs (miRNAs) at about 22 nt are two subtypes of ncRNAs [10–12]. They can affect various biological processes in a molecular mechanism-dependent manner in diseases. For example, H19 activates AAA progression [13]. Interleukin 6 (IL-6) increases the level of NADPH oxidase 2 in human aortic endothelial cells with the assistance of MALAT1 [14]. miR-24 restricts inflammation of aortic vascular and murine abdominal aneurysm [15]. MicroRNA-712 and microRNA-205 prevent AAA in angiotensin II (Ang-II)-infused mice [16]. miR-191-5p is researched to be elevated and promote the development in multiple sclerosis and osteosarcoma [17, 18]. Importantly, miR-191 is reported to be highly expressed in AAA [19]. miR-191-5p of the miR-191 family has also been demonstrated to affect senescence in ascending aortic aneurysm [20]. However, the specific function of miR-191-5p and relevant regulatory mechanism are unknown.

lncRNA MIR503HG is a newly identified repressor in several cancers [21–23]. In this study, we also aimed to figure out whether MIR503HG was involved in the regulatory network of miR-191-5p in AAA.

Herein, AAA patients' tissues and VSMCs were acquired for evaluating gene expressions. Cell viability, cell apoptosis, extracellular matrix (ECM) disruption, and inflammation responses were tested to analyze the functional influence of miR-191-5p. Additionally, mechanism assays were designed for detecting relations between genes.

## 2. Materials and Methods

### 2.1. Patients and Cell Lines

43 AAA patients and controls were recruited for collecting AAA and normal aortic tissues which were resected and snap-frozen at -80°C in liquid nitrogen immediately. Clinical analysis was conducted with the ethical approval from the Research Ethics Committee of Henan Provincial People's Hospital. Informed consent was provided by all participants. Primary human aortic VSMCs were preserved in RPMI-1640 Glutamax with 1% antibiotic solution (10,000 U/mL streptomycin sulphate and 10,000 U/mL penicillin G) and 10% FBS. Additionally, HEK-293T cells were procured from Procell (Wuhan, China) and incubated in DMEM+10% FBS+1% penicillin/streptomycin. The environment was kept at 37°C with 5% CO_2_.

### 2.2. Quantitative Real-Time PCR (qRT-PCR)

TRIzol reagent (Life Technologies, Gaithersburg, MD, USA) was obtained for the extraction of total RNAs from VSMCs. TaqMan Reverse Transcription Kit (Applied Biosystems, Foster City, CA, USA) synthesized the first strand cDNA. Subsequent qPCR was completed using Applied Biosystems Prism 7900HT Fast Real-Time PCR system and SYBR-Green (Applied Biosystems). RNA expression was analyzed by the method of ΔΔCt [24] and normalized to U6 or GAPDH. Primer sequences are listed in Table 1.

### 2.3. Cell Transfection

The transfection procedures were in line with previous study [25]. VSMCs were cultivated until they reached 60-70% confluence. Subsequently, cells (80,000-120,000) in a 6-well culture plates with serum-free DMEM were subjected to transfection with various plasmids at the concentration of 10 nM using Lipofectamine 2000 reagent (Invitrogen, Carlsbad, CA, USA). The miR-191-5p mimics and miR-NC, pcDNA3.1/PLCD1 (PLCD1), and empty vector were provided by GenePharma (Shanghai, China). The miR-191-5p inhibitor and miR-NC, as well as the shRNAs specific to MIR503HG (shMIR503HG), PLCD1 (shPLCD1), and control shRNA (shNC), were also synthesized by GenePharma for gene silencing. 48 h later, VSMCs were harvested. The efficiency was measured by qRT-PCR.

### 2.4. CCK-8 Assay

VSMCs in 96-well plates (1 × 10^4^/well) were transfected with plasmids and respective control. After 48 h, the samples were incubated with CCK-8 kit (Beyotime Institute of Biotechnology, Shanghai, China) for 2 h. The viability of VSMCs was monitored by measuring the OD value at 405 nm.

### 2.5. EdU Assay

EdU incorporation assay kit was procured from RiboBio (Guangzhou, China). Transfected cells were mixed with 100 *μ*L of EdU medium diluent for 3 h in 96-well plates, followed by culture with 100 *μ*L of 1x Apollo^®^ 488 liquid in 4% paraformaldehyde for 30 min. Nuclei were counterstained with DAPI (Beyotime) for observation.

### 2.6. Flow Cytometry of Cell Apoptosis

VSMCs were transfected and collected to the 6-well plates (3 × 10^3^/well), followed by double-stained with Annexin V/PI Kit (BD Biosciences, San Jose, CA, USA). Apoptosis of VSMCs was analyzed by FACSCalibur flow cytometer (BD Biosciences).

### 2.7. TUNEL Assay

Transfected VSMCs were fixed and permeabilized with 1% formaldehyde and 0.2% Triton X-100, respectively. After dUTP-end labeling (Clontech, Mountain View, CA, USA) and DAPI staining, VSMCs were visualized by TE200-U fluorescence microscope (Nikon, Tokyo, Japan).

### 2.8. Caspase-3 Activity Detection

Solarbio (Beijing, China) provided the caspase-3 activity kit for this assay. Protein extracts were cultivated with reaction buffer for 4 h, with the addition of caspase substrate in 96-well dishes. The environment was kept at 37°C. Caspase-3 activity was detected at 405 nm by a microplate reader.

### 2.9. Western Blot

VSMCs in RIPA lysis buffer were loaded onto 10% SDS-PAGE and transferred onto PVDF membranes (Millipore, Bedford, MA, USA) at 80 V, following treatment with 5% nonfat dry milk. The primary antibodies (1 : 1,000) against Ki67, Bcl-2, Bax, Total-caspase-3, Cleaved-caspase-3, MMP2, MMP9, TIMP-1, *α*-SMA, OPN, PLCD1, and GAPDH, as well as secondary antibodies conjugated with HRP (1 : 2,000), were all acquired from Abcam (Cambridge, MA, USA). The protein signals were analyzed by enhanced chemiluminescence (ECL) reagent (GE Healthcare, Milwaukee, MI, USA) following the guidelines.

### 2.10. ELISA Assay

VSMCs were transfected in 96-well plates (1 × 10^4^/well) for 48 h. The culture medium was collected and maintained at -80°C. The cytokine TNF-*α* or IL-6 levels were assessed by Human TNF-*α* or IL-6 Quantikine ELISA Kit (R&D Systems, Minneapolis, MN, USA).

### 2.11. RNA Pull-Down Assay

The miR-191-5p and its antisense RNA (miR-191-5p AS) were in vitro biotin-labeled for obtaining the Bio-miR-191-5p sense and Bio-miR-191-5p AS. Cell lysates were incubated with biotinylated RNAs and Bio-NC. The pull-down complex was analyzed by qRT-PCR.

### 2.12. Dual-Luciferase Reporter Analysis

The wild-type (WT) or mutant (Mut) miR-191-5p binding sites to MIR503HG sequence were inserted into pmirGLO Dual-Luciferase Vector (Promega, Madison, WI, USA) and named as MIR503HG-WT/Mut. VSMCs and HEK-293T were transfected with MIR503HG-WT/Mut in the presence of miR-191-5p mimics and miR-NC for 48 h. The 3′UTRs of 13 possible mRNAs within predicted interacting sequences of miR-191-5p were separately cloned into pmirGLO Vectors and cotransfected with miR-191-5p mimics or miR-NC into VSMCs and HEK-293T. After transfection, Dual-Luciferase Reporter Assay System (Promega) was utilized for determination of luciferase activity.

### 2.13. Statistical Analyses

The data were shown as mean ± SD with at least three replications. Two-tailed Student's *t*-test or ANOVA by use of SPSS version 19.0 (SPSS, Chicago, IL, USA) was utilized for statistical analyses. A *P* value less than 0.05 was considered as the threshold value. Spearman's correlation analysis analyzed the correlation between every two genes.

## 3. Results

### 3.1. miR-191-5p Negatively Regulated the Apoptosis, ECM Degradation, and Inflammation in AAA

**Figure 1:**
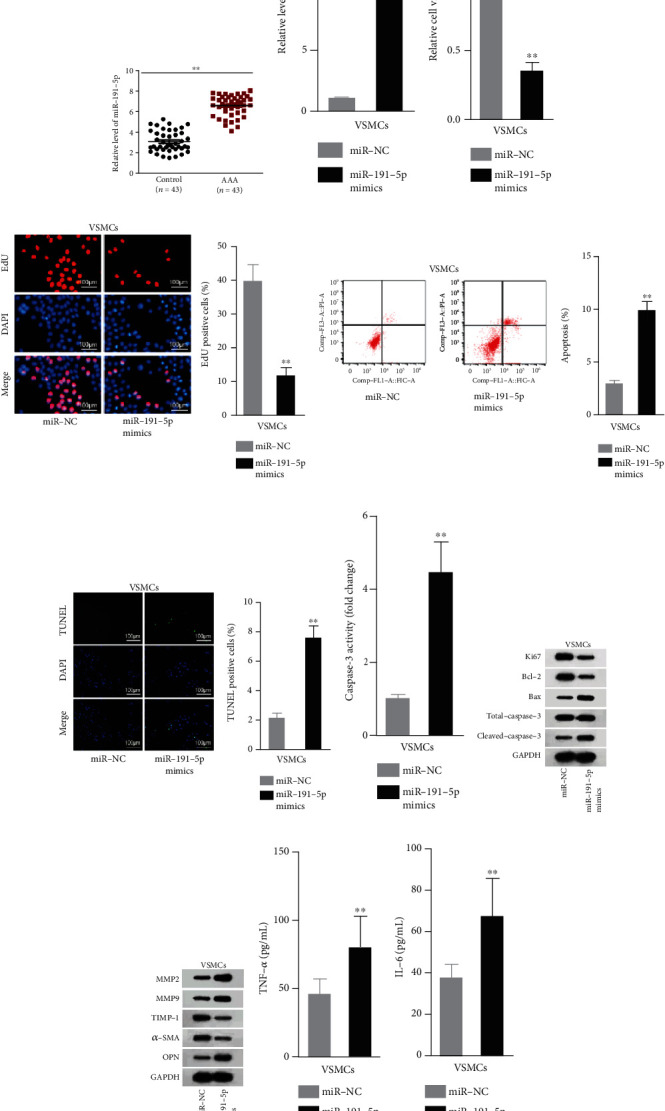
miR-191-5p augment influenced behaviors of VSMCs in AAA. (a) AAA tissues and control tissues were obtained for measurement of miR-191-5p level through qRT-PCR. (b) qRT-PCR data revealed the overexpression efficiency of miR-191-5p mimics in VSMCs. (c, d) CCK-8 and EdU staining assays were conducted for detecting the viability of VSMCs under miR-191-5p overexpression. (e–g) miR-191-5p mimic influence on VSMC apoptosis was assayed via flow cytometry, TUNEL staining, and caspase-3 activity detection assays. (h, i) Western blotting for the proliferation/apoptosis-associated proteins and proteins involved in ECM degradation after miR-191-5p mimic transfection. (j, k) Serum levels of the TNF-*α* and IL-6 were quantified by ELISA kits in VSMCs. Three biological replicates were involved for each experiment. ^∗∗^*P* < 0.01.

miR-191-5p is demonstrated to be elevated in aortic aneurysm, but its function is not explored in detail [20]. Therefore, we first detect the expression of miR-191-5p in clinical samples. As a result, miR-191-5p was significantly elevated in AAA tissues in comparison with controls (Figure 1(a)). For confirming the participation of miR-191-5p during AAA development, we carried out gain-of-function experiments with VSMCs. In preparation, miR-191-5p expression was elevated in VSMCs (Figure 1(b)). Then, we detected that when miR-191-5p was enhanced, cell viability and proliferation were dramatically reduced (Figures 1(c) and 1(d)). In addition, experimental results also demonstrated that cell apoptosis was induced under miR-191-5p increment (Figures 1(e)–1(g)). Subsequently, western blot affirmed that Ki67 and Bcl-2 proteins declined, but Total-caspase-3 protein was not changed, and the Bax and Cleaved-caspase-3 proteins were augmented, hinting that cell apoptosis was accelerated (Figure 1(h)). The impact of miR-191-5p elevation on ECM degradation was then analyzed. From western blot results, augmented miR-191-5p expression enhanced MMP2 and MMP9 protein expressions (enzymes able to degrade various components of ECM proteins [26]) but lessened the expression level of their inhibitor TIMP-1. The protein levels of *α*-SMA, a marker of contractile VSMCs, were downregulated. The protein levels of OPN, a marker of synthetic VSMCs, were upregulated (Figure 1(i)). Additionally, ELISA kit detected the augment of TNF-*α* and IL-6, two proinflammatory mediators (Figures 1(j) and 1(k)). Taken together, miR-191-5p elevation could cause the promotion of apoptosis, ECM degradation, and inflammation.

### 3.2. MIR503HG Bound with miR-191-5p in VSMCs

**Figure 2:**
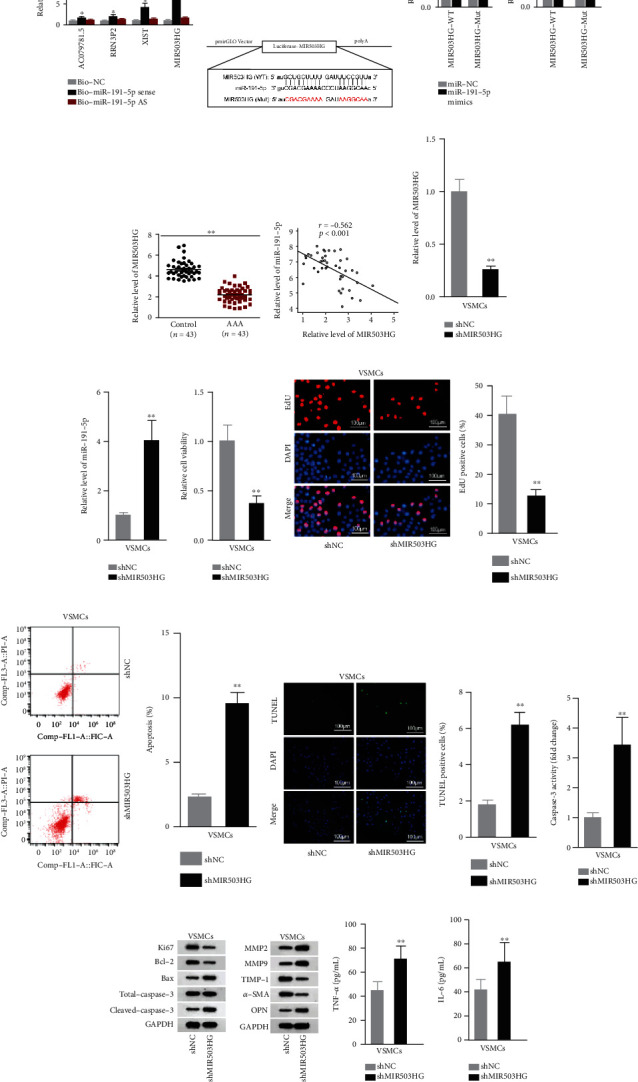
MIR503HG sponged miR-191-5p in VSMCs. (a) Pull-downs of biotinylated probes were quantified by qRT-PCR. (b, c) miR-191-5p binding sites on MIR503HG sequence (wild or mutant) were cloned into pmirGLO Vector for luciferase reporter assays. (d) MIR503HG expression in AAA tissues versus control samples was shown. (e) MIR503HG and miR-191-5p expression correlation was determined by Spearman's correlation analysis. (f) qRT-PCR was done for the silencing efficiency of shMIR503HG. (g) The miR-191-5p level in response to MIR503HG depletion was analyzed. (h, i) VSMC viability responding to shMIR503HG transfection was tested. (j–l) VSMC apoptosis after shMIR503HG transfection was analyzed. (m, n) The levels of proliferation/apoptosis-associated proteins and proteins involved in ECM degradation after silencing MIR503HG were evaluated. (o, p) Serum levels of inflammatory cytokines in shMIR503HG-regulated VSMCs were analyzed. Three biological replicates were involved for each experiment. ^∗^*P* < 0.05 and ^∗∗^*P* < 0.01.

To figure out the lncRNA targeting miR-191-5p, starBase was applied for prediction. Four possible lncRNAs binding with miR-191-5p were found, namely, AC079781.5, RRN3P2, XIST, and MIR503HG. We noticed that MIR503HG exhibited the highest enrichment in the Bio-miR-191-5p sense group, compared to the control group and Bio-miR-191-5p antisense group (Figure 2(a)). Thus, MIR503HG was tentatively selected. Thereafter, we constructed pmirGLO Vectors containing wild-type and mutant miR-191-5p binding sequences in MIR503HG, which are shown in Figure 2(b). The luciferase activity of MIR503HG-WT was markedly impaired by the increment of miR-191-5p (Figure 2(c)). Then, low MIR503HG expression was found in AAA tissues, which could negatively modulate miR-191-5p expression, as measured by qRT-PCR and Spearman's correlation analysis (Figures 2(d) and 2(e)). Consistently, we found that in VSMCs, miR-191-5p expression was promoted by MIR503HG downregulation (Figures 2(f) and 2(g)). The impacts of MIR503HG on VSMCs were also explored. As a result, cell viability and proliferation were hampered when MIR503HG was silenced (Figures 2(h) and 2(i)). Additionally, experimental results also validated that MIR503HG inhibition enhanced the cell apoptosis (Figures 2(j)–2(m)). ECM degradation and inflammation were also enhanced by the depletion of MIR503HG (Figures 2(n)–2(p)). Notably, MIR503HG interacted with miR-191-5p to promote the proliferation of VSMCs while impeding apoptosis, slowing down ECM degradation, and inhibiting inflammation.

### 3.3. PLCD1 Was the Downstream Target Underlying MIR503HG

**Figure 3:**
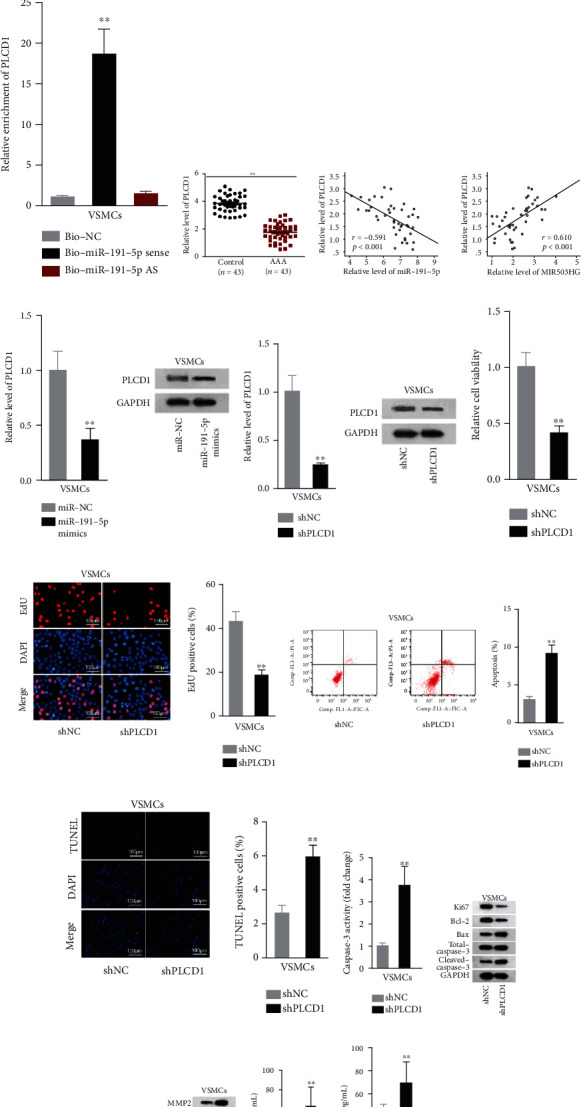
miR-191-5p targeted PLCD1 in VSMCs. (a) 13 possible mRNAs of miR-191-5p were screened out and presented as the Venn diagram. (b) Change in luciferase activity revealed the interplay of miR-191-5p and 13 possible mRNAs. (c) PLCD1 enrichment in pull-down complex by biotinylated miR-191-5p was measured. (d) PLCD1 levels in AAA tissues compared to the control group were measured. (e) Correlation between expressions of PLCD1 and miR-191-5p or MIR503HG was analyzed. (f, g) PLCD1 level in differentially transfected VSMCs was analyzed. (h–l) The regulation of PLCD1 knockdown on VSMC viability and apoptosis was assessed. (m, n) The levels of proliferation/apoptosis-associated proteins and proteins involved in ECM degradation under PLCD1 depletion were assessed. (o, p) Serum levels of inflammatory cytokines in shPLCD1-regulated VSMCs were measured. Three biological replicates were involved for each experiment. ^∗∗^*P* < 0.01.

To determine the downstream mRNA of miR-191-5p, after the prediction from microT, miRanda, miRmap, PicTar, PITA, and TargetScan, thirteen mRNAs were selected for further investigations (Figure 3(a)). Then, we found that the luciferase activity of PLCD1 3′UTR was most restrained by miR-191-5p elevation (Figure 3(b)). Moreover, PLCD1 was largely pulled down by Bio-miR-191-5p sense probe but not by Bio-NC or Bio-miR-191-5p antisense probe (Figure 3(c)). In addition, qRT-PCR analysis indicated that PLCD1 levels were relatively low in AAA tissues (Figure 3(d)). The negative association between PLCD1 and miR-191-5p, as well as the positive association between PLCD1 and MIR503HG, was demonstrated through Spearman's correlation analysis (Figure 3(e)). Thus, PLCD1 was confirmed as the mRNA target of miR-191-5p. PLCD1 expression was declined at both mRNA and protein levels by miR-191-5p elevation (Figure 3(f)). Subsequently, PLCD1 was knocked down via shPLCD1 transfection (Figure 3(g)). Inhibited PLCD1 refrained cell proliferation and facilitated cell apoptosis, ECM degradation, and inflammation in VSMCs (Figures 3(h)–3(p)). Collectively, miR-191-5p targeted PLCD1, which could enhance the proliferation but prevent the apoptosis, ECM degradation, and inflammation.

### 3.4. Upregulation of PLCD1 Countered the Impacts of miR-191-5p Increase on the Cellular Activities of VSMCs

**Figure 4:**
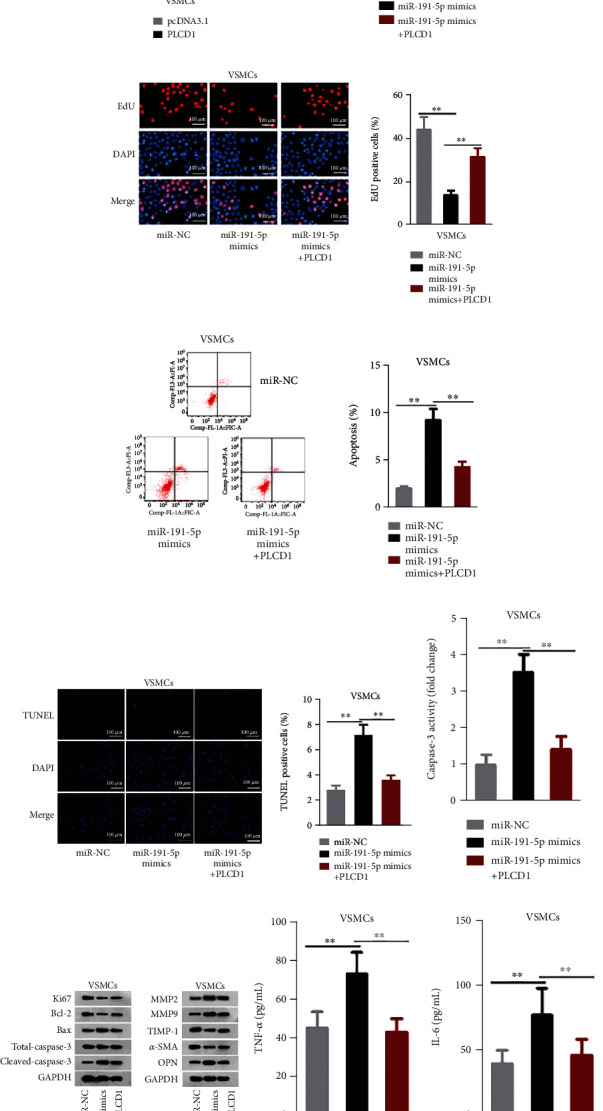
PLCD1 overexpression weakened miR-191-5p mimic-mediated AAA progression. (a) The overexpression efficiency of pcDNA3.1/PLCD1 (PLCD1) in VSMCs was evaluated. (b–f) The viability and apoptosis in three groups of differentially transfected VSMCs were analyzed. (g, h) The levels of proliferation/apoptosis-related proteins and proteins related to ECM degradation in each group were tested. (i, j) Serum levels of inflammatory cytokines in each sample. Three biological replicates were involved for each experiment. ^∗∗^*P* < 0.01.

To testify the regulatory function of miR-191-5p/PLCD1 axis in VSMCs, rescue assays were carried out. PLCD1 was overexpressed by PLCD1 overexpression vectors (Figure 4(a)). In CCK-8 and EdU assays, the miR-191-5p mimic-inhibited viability was rescued by upregulating PLCD1 (Figures 4(b) and 4(c)). As examined by flow cytometry, TUNEL, and caspase-3 activity kit, miR-191-5p promotion-accelerated apoptosis was reversed by elevation of PLCD1 (Figures 4(d)–4(f)). Western blot reconfirmed these results since the protein levels of Ki67 and Bcl-2 were lessened when miR-191-5p was elevated but reversed when PLCD1 was added. Bax and Cleaved-caspase-3 were augmented when miR-191-5p was overexpressed but recovered when PLCD1 was upregulated (Figure 4(g)). As for ECM, it was observed that miR-191-5p enhancement improved the protein levels of MMP2, MMP9, and OPN and lowered the protein levels of TIMP-1 and *α*-SMA, which was later neutralized by transfection of PLCD1 overexpression plasmids (Figure 4(h)). Besides, inflammation response facilitated by miR-191-5p increase was hindered by PLCD1 upregulation (Figures 4(i) and 4(j)). In conclusion, miR-191-5p exerted its function in VSMCs through PLCD1.

### 3.5. MIR503HG/miR-191-5p/PLCD1 Pathway Played an Important Part in the AAA Development

**Figure 5:**
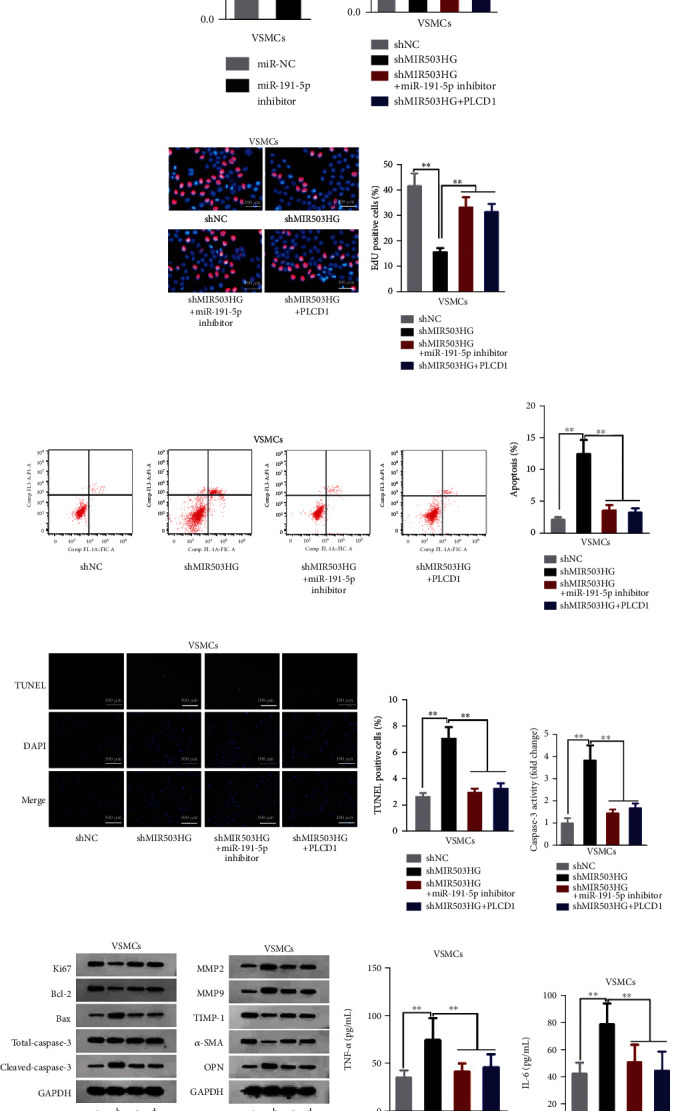
MIR503HG influenced AAA progression via miR-191-5p/PLCD1. (a) qRT-PCR assessed miR-191-5p inhibitor efficiency in VSMCs. (b–f) The viability and apoptotic capacities of VSMCs in four differently transfected groups were analyzed. (g, h) The levels of proliferation/apoptosis-correlated proteins and proteins associated with ECM degradation in each treatment group were detected. (i, j) Serum levels of the inflammatory cytokines in 4 groups were quantified. Three biological replicates were involved for each experiment. ^∗∗^*P* < 0.01.

Subsequently, the function of MIR503HG/miR-191-5p/PLCD1 axis was validated. Before rescue experiments, miR-191-5p was silenced through transfection of miR-191-5p inhibitor (Figure 5(a)). After conducting a series of assays, we confirmed that cell viability weakened by MIR503HG knockdown was facilitated by miR-191-5p silence or PLCD1 enhancement (Figures 5(b) and 5(c)), while cell apoptosis activated due to MIR503HG repression was impaired with miR-191-5p depletion or PLCD1 increment, as determined by flow cytometry, TUNEL, and caspase-3 activity kit (Figures 5(d)–5(f)). We also detected that the reduction in Ki67 and Bcl-2 proteins and increase in Bax and Cleaved-caspase-3 proteins by MIR503HG silencing were abrogated by miR-191-5p inhibitor or PLCD1 (Figure 5(g)). Additionally, ECM degradation and inflammation boosted by depletion of MIR503HG were offset by knockdown of miR-191-5p or addition of PLCD1. In the shMIR503HG group, protein levels of MMP2, MMP9, and OPN as well as TNF-*α* and IL-6 levels were augmented, and protein levels of TIMP-1 and *α*-SMA were lowered compared with the control group. These phenomena were counteracted by cotransfection of miR-191-5p inhibitor or of PLCD1 (Figures 5(h)–5(j)). In summary, MIR503HG negatively mediated the apoptosis, ECM degradation, and inflammation in VSMCs via miR-191-5p/PLCD1.

## 4. Discussion

The apoptosis and phenotypic shift of VSMCs and inflammation response are noted features of AAA. More apoptotic and synthetic VSMCs as well as induced inflammation are observed in AAA progression. The apoptotic VSMCs result in the numerous loss of contractile cells and thus promoting the expansion of the abdominal aortic wall [27]. Furthermore, it has been revealed that inflammation could secrete MMP, which can further degrade the components of the ECM and cause ECM disruption. The TIMP family has been documented to strictly regulate MMP family members [28]. Also, a synthetic phenotype of VSMCs occurs due to the imbalance of synthetic and contractile phenotype. *α*-SMA is a biomarker of contractile VSMCs, and OPN is a biomarker of synthetic VSMCs. In this study, all these factors were tested to reflect the functions of genes in AAA.

Past studies have excavated that miR-191-5p was elevated in aortic aneurysm, but its function role in AAA has never been explored [19, 20]. Hence, we attempted to explicitly figure out the role of miR-191-5p in AAA. In this paper, elevated miR-191-5p expression was discovered in AAA tissues. In addition, miR-191-5p promotion repressed cell viability, enhanced cell apoptosis, and enhanced ECM disruption and inflammation in VSMCs. This was the first time that miR-191-5p was demonstrated as a contributor for AAA. From starBase, we obtained four lncRNAs targeting miR-191-5p. MIR503HG was the most possible lncRNA binding to miR-191-5p in VSMCs, which was downregulated in AAA tissues and had negative regulation on miR-191-5p. In consistent with the antioncogenic role of MIR503HG played in tumors [21–23], the role of MIR503HG in VSMCs was proven to be suppressive. Then, we continued to probe into the downstream factor that MIR503HG/miR-191-5p axis targeted in VSMCs. Among the thirteen targets gained from the Venn diagram, pPLCD1 was screened out for its significantly declined luciferase activity under the improvement of miR-191-5p. PLCD1 was previously disclosed to inhibit diseases, airway smooth muscle hypertrophy, colorectal cancer, and breast cancer contained [29–31]. PLCD1 as a target of miR-191-5p was affirmed through mechanism experiments. PLCD1 also exerted suppressive function in VSMCs. The functions of miR-191-5p/PLCD1 axis and MIR503HG/miR-191-5p/PLCD1 axis in VSMCs were verified via rescue assays.

Totally, in AAA progression, the sponge of miR-191-5p by MIR503HG was weakened, and therefore, PLCD1 expression was decreased, resulting in weakened viability, facilitated apoptosis, and enhanced ECM disruption and inflammation. Addition of MIR503HG might be a novel therapeutic strategy for patients with AAA.

## Figures and Tables

**Table 1:** Primer sequences used in qRT-PCR were listed.

Primer sequences used in qRT-PCR			
Gene name	Primer sequence	Company name	Location
miR-191-5p	Reverse transcription stem loop primer: CTCAACTGGTGTCGTGGAGTCGGCAATTCAGTTGAGCcagctgct miRNA stem loop universal reverse primer: CTCAACTGGTGTCGTGGA F: GCCGAGtttgggattccgttg	NCBI	USA

AC079781.5	F: CGTGTCCAGAATTGGTGGGT R: GCACCCTGTCAAAACACACC	primer3plus	

RRN3P2	F: TCTGCTTGCGGTTGGATAGC R: TCTCCAGCAAACTGAGCCAC	NCBI	USA

XIST	F: TTAAAGCGCTGCAATTCGCT R: AGGGTGTTGGGGGACTAGAA	NCBI	USA

MIR503HG	F: TCCCGCCAAATGAGTCAGTC R: ACAGAGTTGTGACCACTGCC	NCBI	USA

PLCD1	F: GGACTTCCTGACCCTGCAC R: TTCGCACCTCCTGAATGTCC	NCBI	USA

## Data Availability

The data used to support the research findings are included within this article.
